# Leucine-Enriched Essential Amino Acids Improve Recovery from Post-Exercise Muscle Damage Independent of Increases in Integrated Myofibrillar Protein Synthesis in Young Men

**DOI:** 10.3390/nu12041061

**Published:** 2020-04-11

**Authors:** Marcus Waskiw-Ford, Sarkis Hannaian, Justin Duncan, Hiroyuki Kato, Sidney Abou Sawan, Marius Locke, Dinesh Kumbhare, Daniel Moore

**Affiliations:** 1 Faculty of Kinesiology and Physical Education, University of Toronto, Toronto, ON M5S 2C9, Canada; marcus.waskiwford@mail.utoronto.ca (M.W.-F.); sarkis.hannaian@mail.utoronto.ca (S.H.); justin.duncan@mail.utoronto.ca (J.D.); sidney.abousawan@mail.utoronto.ca (S.A.S.); marius.locke@utoronto.ca (M.L.); 2 Technology Development Center, Institute of Food Sciences and Technologies, Ajinomoto Co., Inc., Kawasaki, Kanagawa 210-8681, Japan; hiroyuki_kato@ajinomoto.com; 3 Toronto Rehabilitation Institute, Toronto, ON M5G 2A2, Canada; dinesh.kumbhare@uhn.ca

**Keywords:** amino acids, resistance exercise, muscle protein synthesis, muscle damage, recovery, strength

## Abstract

Background: Leucine-enriched essential amino acids (LEAAs) acutely enhance post-exercise myofibrillar protein synthesis (MyoPS), which has been suggested to be important for muscle repair and recovery. However, the ability of LEAAs to concurrently enhance MyoPS and muscle damage recovery in free-living humans has not been studied. Methods: In a randomized, double-blind, placebo-controlled, parallel-group design, twenty recreationally active males consuming a controlled diet (1.2 g/kg/d of protein) were supplemented thrice daily with 4 g of LEAAs (containing 1.6 g leucine) or isocaloric placebo for four days following an acute bout of lower-body resistance exercise (RE). MyoPS at rest and integrated over 96 h of recovery was measured by D_2_O. Isometric and isokinetic torque, muscle soreness, Z-band streaming, muscle heat shock protein (HSP) 25 and 72, plasma creatine kinase (CK), and plasma interleukin-6 (IL-6) were measured over 96 h post-RE to assess various direct and indirect markers of muscle damage. Results: Integrated MyoPS increased ~72% over 96 h after RE (*p* < 0.05), with no differences between groups (*p* = 0.98). Isometric, isokinetic, and total peak torque decreased ~21% by 48 h after RE (*p* < 0.05), whereas total peak torque was ~10% greater overall during recovery in LEAAs compared to placebo (*p* < 0.05). There were moderate to large effects for peak torque in favour of LEAAs. Muscle soreness increased during recovery with no statistical differences between groups but small to moderate effects in favour of LEAAs that correlated with changes in peak torque. Plasma CK, plasma IL-6, and muscle HSP25 increased after RE (*p* < 0.05) but were not significantly different between groups (*p* ≥ 0.13). Consistent with a trend toward attenuated Z-band streaming in LEAAs (*p* = 0.07), muscle HSP72 expression was lower (*p* < 0.05) during recovery in LEAAs compared with placebo. There were no correlations between MyoPS and any measures of muscle damage (*p* ≥ 0.37). Conclusion: Collectively, our data suggest that LEAAs moderately attenuated muscle damage without concomitant increases in integrated MyoPS in the days following an acute bout of resistance exercise in free-living recreationally active men.

## 1. Introduction

Muscle protein synthesis (MPS) is elevated for up to 48 h after resistance exercise (RE) as a means to remodel and repair skeletal muscle [1,2,3]. It is well established that acute essential amino acid (EAA) ingestion amplifies the anabolic effect of RE on MPS in traditional laboratory based studies [4,5,6]. Among the EAAs, leucine is thought to be the primary stimulator of MPS through its ability to activate the mammalian target of the rapamycin complex 1 (mTORC1) pathway and, when ingested or infused in large doses, independently stimulates MPS [7,8,9]. However, in the absence of an adequate quantity of the other EAAs, leucine alone may not be sufficient to support maximal rates of MPS [10]. Thus, providing leucine-enriched essential amino acids (LEAAs) has been shown to augment rates of MPS in the hours following exercise in controlled laboratory settings [11,12,13,14,15,16,17]. However, these studies generally do not capture the complete post-exercise recovery period, nor consider whether supplementation within the context of a complete diet may also be efficacious. Although there is some evidence in older adults that the leucine content of a diet may influence ‘free-living’ rates of myofibrillar protein synthesis (MyoPS) after exercise [18,19], there is limited research on the impact of LEAAs in younger populations consuming a moderate protein diet.

Unaccustomed RE results in structural perturbations of the muscle cytoskeletal architecture (e.g., Z-band streaming) that is commonly characterized as muscle damage [20,21], although these have also been suggested to represent focalized areas of myofibrillar remodeling [22]. Acute structural damage can lead to cell stress and the upregulation of heat shock proteins (HSP, e.g., HSP25, HSP72), which can function as molecular chaperones to prevent the folding, aggregation, and/or degradation of damaged proteins [23]. These ‘direct’ markers of muscle damage typically present 24–72 h after exercise [20,24], suggesting that the post-exercise increase in MyoPS may serve to repair and/or attenuate structural muscle damage [2]. Accordingly, LEAAs may represent an appropriate nutritional approach to accelerate the recovery from exercise-induced muscle damage. However, to date, we are not aware of any studies that have concurrently determined whether the nutritional regulation of MyoPS aligns with the presence or resolution of markers of structural damage after a bout of novel exercise.

Muscle damage induced by unaccustomed RE is also commonly assessed through surrogate markers including loss of force [20], increased muscle soreness [25], and elevated plasma creatine kinase (CK) [26]. In this context, protein and amino acid supplementation may have the potential to attenuate the extent of exercise-induced muscle damage as evaluated by surrogate markers following novel strenuous RE [27,28]. For example, LEAAs were more effective than placebo in restoring muscle force production [29] and alleviating muscle soreness (evaluated by paw withdrawal threshold) [12] in the days following eccentric contractions in rodents. In studies involving eccentric knee extension exercise in untrained males, supplementation with branched-chain amino acids (BCAAs) reduced muscle soreness [30], whereas whey protein supplementation [31] preserved muscle force. Furthermore, improved plasma CK levels were observed with LEAA supplementation following an acute bout of repeated elbow flexion and extension in untrained males [32]. However, similar to ‘direct’ measures of muscle damage, no study to our knowledge has systematically examined the potential relationship between MyoPS and these common indirect (e.g., soreness) or performance (e.g., strength) markers of muscle damage in response to nutritional intervention, which has been highlighted previously as a limitation [27].

Accordingly, the purpose of this study was to determine the impact of LEAA supplementation on muscle recovery over a 96 h post-RE period in free-living conditions through assessments of integrated MyoPS and various markers of muscle damage and performance. This allowed us to investigate the relationship between integrated MyoPS and muscle damage recovery following RE. We hypothesized that the provision of LEAAs would increase post-RE integrated 96 h MyoPS compared to an isocaloric placebo, and that this would be associated with reductions in muscle damage outcomes.

## 2. Materials and Methods

### 2.1. Participants

Twenty recreationally active and healthy young men (Table 1) provided written consent to participate in a protocol that was written in accordance with standards set by the revised (2008) Declaration of Helsinki and approved by the research ethics board at the University of Toronto, Canada (protocol #34365) and the institutional review board of Ajinomoto Co., Inc (No. 2016–036). This study was pre-registered at https://www.clinicaltrials.gov as NCT03319147. Participants completed a Physical Activity Readiness Questionnaire [33] and the International Physical Activity Questionnaire [34] to verify that they could complete the RE protocol, and were excluded if they had performed lower-body resistance training six months prior to enrollment. After an overnight fast and prior to any water consumption, air displacement plethysmography (BOD−POD, COSMED USA Inc., Chicago, IL, USA) was used to characterize body composition and estimate resting energy expenditure (REE). At least four weeks prior to the exercise trial, one repetition maximum (1RM) and habitual diet data were obtained, and participants were familiarized with maximal voluntary contraction assessments.

### 2.2. Exercise Trial

**Figure 1:**
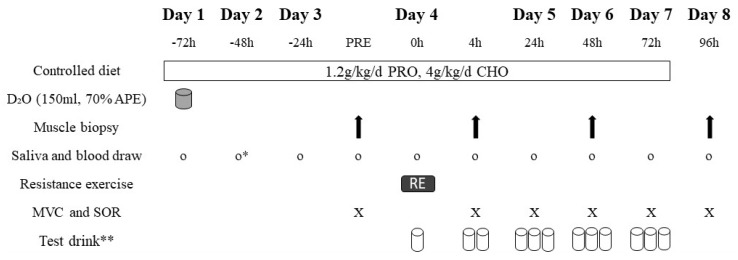
Overview of the 8 day exercise trial. PRE, pre-exercise; PRO, protein; CHO, carbohydrate; D_2_O, deuterium oxide; MVC, maximal voluntary contraction; SOR, perceived muscle soreness. * No blood draw was performed on Day 2. ** Test drink consisted of LEAAs or placebo. First test drink on Day 4 was consumed immediately after resistance exercise, second test drink on Day 4 was consumed at 4 h after all measurements, and third test drink on Day 4 was consumed at least three hours later.

In a randomized, double-blind, placebo-controlled, parallel-group design, participants underwent an 8 day exercise trial after being assigned to the LEAA or placebo group. Participants were instructed not to perform any structured physical activity during the 8 day trial. Daily physical activity was tracked via accelerometer (wGT3X−BT, Actigraph, Pensacola, FL, USA), which allowed us to confirm that accelerometer−based measures of total energy expenditure, moderate to vigorous physical activity, and total step count were not different between groups. An overview of the 8 day exercise trial is outlined in Figure 1. On Day 1, a fasted blood sample from an antecubital vein and an oral saliva sample were obtained to determine baseline body water enrichment. Immediately following baseline blood and saliva sampling, participants ingested a 150 mL oral bolus of 70% deuterated water (D_2_O). A second saliva sample was obtained two hours following the ingestion of D_2_O. A fasted saliva sample was obtained on Day 2, and both fasted saliva and blood samples were obtained on Day 3.

On Day 4, while fasted, a baseline muscle biopsy was obtained from the non-dominant leg along with blood and saliva samples. A standardized breakfast (0.3 g/kg protein, 1.0 g/kg carbohydrate, and total calories equivalent to 0.4*REE) was consumed immediately post-biopsy, and a minimum of one hour elapsed post-breakfast before participants underwent the RE protocol. Saliva and blood samples were obtained directly post-RE, and then participants immediately ingested either a LEAA or placebo drink (time 0 h). A second muscle biopsy from the same leg was obtained at 4 h post-RE, along with saliva and blood samples. On Days 5–8, fasted saliva and blood samples were obtained at 24, 48, 72, and 96 h post-RE, whereas third and fourth muscle biopsies from the same leg as the previous biopsies were obtained at 48 and 96 h post-RE, respectively. This unilateral biopsy approach was designed to minimize any biopsy induced discomfort from impacting our secondary outcomes of muscle strength and soreness (see below). In addition, as acute, low-load exercise can stimulate muscle protein synthesis [35], the completion of the repeated post-exercise performance tests could have impacted our myofibrillar protein synthetic and HSP responses and therefore influenced our measures of physiological recovery.

#### 2.2.1. Measurements of Muscle Strength and Soreness

Maximal muscle strength and muscle soreness were assessed pre-RE and 4, 24, 48, 72, and 96 h post-RE on the contralateral leg from which biopsies were obtained. Maximal muscle strength was assessed by maximal voluntary isometric contraction and isokinetic shortening contractions (MVC) at slow (60°/s) and fast (270°/s) angular velocities using an isokinetic dynamometer (Biodex Medical Systems Inc., Shirley, New York, USA) [36]. Each contraction type was attempted three times with sixty seconds of rest in between, and the highest peak torque obtained among the three attempts was considered as the MVC value. Total peak torque was calculated as the summation of isometric, slow isokinetic, and fast isokinetic MVC maneuvers.

Subjective ratings of muscle soreness (thigh, hamstring, and calf) were collected from participants using a 100 mm visual analogue scale. Extremes of 0 and 100 mm were anchored as “no pain” and “worst possible pain”, respectively, and participants drew a line on the scale indicating their perceived soreness. Total perceived muscle soreness was calculated as the sum of thigh, hamstring, and calf muscle soreness.

#### 2.2.2. Controlled Diet

Participants were on a controlled diet throughout the duration of the exercise trial in the form of commercially available, pre-packaged foods that provided energy to cover 1.6*REE. The macronutrient distribution was 1.2 g/kg/d of protein, 4 g/kg/d of carbohydrate, and the remaining calories from fat. Although this protein intake is slightly lower than the habitual dietary intake of our participants (Table 1) and the estimated population average of North America [37], it is sufficient for recreationally active males [38]. Participants were instructed not to consume anything other than water outside of the prepared meals and snacks (calorie-free coffee or tea were permitted only after laboratory visits).

#### 2.2.3. Test Drink Composition

Test drinks were consumed three times per day on Days 4−7 and subjects were informed to separate meals and test drinks by at least 1.5 h. LEAA drinks were prepared individually using crystalline amino acids (Ajinomoto North America, Inc., Raleigh, NC, USA) and the placebo drink was energy matched with carbohydrates from Polycal (Nutricia, Utrecht, Netherlands). The LEAA mixture consisted of 4 g of essential amino acids (2% histidine, 11% isoleucine, 40% leucine, 17% lysine, 3%, methionine, 7% phenylalanine, 9% threonine, 1% tryptophan, and 11% valine). In total, 20 g of Tang (Kraft, Mississauga, Canada), 5 g of Splenda (Heartland Consumer Products, Carmel, USA), and 2 g of PFD-1 (Mead Johnson, Zeeland, USA) were added to all drinks to mask the taste and ensure blinding.

#### 2.2.4. Resistance Exercise

The RE protocol was composed of 45° inclined bi-lateral leg press and leg extension. Each exercise consisted of five sets of 9–12 maximum bilateral repetitions at 75% 1RM with 90 s of rest between sets [2].

### 2.3. Measurement of Muscle Variables

Muscle samples were obtained from the vastus lateralis of the non-dominant leg only though percutaneous muscle biopsy with manual suction [39]. The procedure involved administration of a local anaesthetic (2% Xylocaine) prior to a small incision from which muscle was collected. Muscle tissue was dissected free of blood and connective tissue and immediately frozen in liquid nitrogen and stored at −80 °C until analysis. A large piece of the muscle sample (40–50 mg) was used for MyoPS quantification and HSP analysis, whereas a smaller portion was immediately fixed in 2% glutaraldehyde for electron microscopy analysis of Z-band streaming.

#### 2.3.1. Myofibrillar Fractional Synthetic Rate

MyoPS was determined by oral D_2_O ingestion to assess changes in myofibrillar fractional synthesis rates (FSR) integrated over 96 h post-RE [3]. Muscle samples were prepared (isolation, hydrolyzing, purification, and derivatization of the myofibrillar proteins) following procedures as previously described [40,41]. Muscle preparations were analyzed by Metabolic Solutions for incorporation of deuterated alanine with a Thermo Finnigan Delta V IRMS coupled to a Thermo Trace GC Ultra with a GC pyrolysis interface III and Conflow IV (gas chromatograph (GC)-pyrolysis-isotope ratio mass spectrometer (IRMS)) (Metabolic Solutions, Nashua, NH, USA). Body water enrichment following D_2_O ingestion was determined through isotope exchange to acetone using GC−MS as previously described [42]. The fractional synthetic rates of myofibrillar protein (MyoPS) were determined from the incorporation of deuterium−labelled alanine into protein, using the enrichment of body water (corrected for the mean number of deuterium moieties incorporated per alanine, 3.7, and the dilution from the total number of hydrogens in the derivative) as the surrogate precursor labelling between subsequent biopsies [3,43]. In brief, the standard equation was used:

FSR(% × h^−1^) = E_Ala_/(E_BW_ × t) × 3.7 × 100

where E_Ala_ is deuterium enrichment of protein-bound alanine, E_BW_ is mean deuterium precursor enrichment of body water between time points, and t is the time (h) between biopsies. The single biopsy approach was used to estimate resting (baseline) MyoPS [44].

#### 2.3.2. Muscle Heat Shock Protein

Muscle HSP was assessed by Western blotting for muscle HSP25 and HSP72 content using primary polyclonal antibodies specific to muscle HSP25 (ADI-SPP-715, Enzo, Farmingdale, USA) and muscle HSP72 (ADI−SPA−812, Enzo, Farmingdale, USA), diluted 1:1000 in TBST and 2% milk, with secondary antibody diluted 1:20,000 in 3% milk. The overall Western blotting protocol has been previously described [45].

#### 2.3.3. Z-Band Streaming

After being frozen, muscle tissue samples were fixed in osmium tetroxide, dehydrated in graded ethanol baths and embedded in epoxy resin (Spurr’s) with fibers oriented longitudinally. Each block was sectioned (0.5 mm) and marked with toluidine blue. Individual fibers were mainly examined at ×1000 magnification (some samples were examined at up to ×10,000 magnification) by electron microscopy for areas of Z-band streaming and images were obtained. Z-band streaming was scored by analyzing fibers for the percentage area occupied by Z-band streaming per total fiber area in the image, as well as the percentage of fibers containing any Z-band streaming [2]. A second researcher vetted analyzed images to ensure consistency in Z-band streaming scores.

### 2.4. Measurement of Plasma Variables

Blood samples were withdrawn into tubes containing ethylene diamine tetraacetic acid (EDTA) disodium salt and centrifuged for 10 min at 2500 rpm. Plasma was subsequently aliquoted and stored at −80 °C.

#### Plasma Analysis

Quantification of plasma CK activity was performed with a commercial kit (MAK116, Sigma-Aldrich, St. Louis, USA) and quantification of plasma IL-6 concentration was performed with a commercial ELISA kit (Human IL-6 Quantikine, R&D Systems, Minneapolis, USA) in accordance with the manufacturer’s instructions.

### 2.5. Statistical Analysis

Witard et al. [46] reported a difference in MyoPS between 0 and 20 g of whey protein after an acute bout of RE, with rates (mean ± SD) of 0.051 ± 0.014%/h and 0.070 ± 0.14%/h, respectively (n = 12 in each group). Based on these results and with α = 0.05 and β = 0.8, we calculated n = 7 per group would be sufficient to detect differences in 4 h MyoPS between the LEAA and placebo groups. Therefore, we recruited n = 10 per group to account for dropouts and increase power for secondary outcomes. Although the study was powered to detect a difference in 4 h MyoPS based on previous applications of this tracer [43], we were unfortunately unable to obtain physiological rates of 4 h MyoPS and therefore these data were not included.

All variables were examined by two-way ANOVA with treatment and time as factors. When a significant main effect or interaction was observed, differences were examined by Sidak’s multiple comparisons test. Independent samples *t*-tests were used to compare participant characteristics between groups. Due to approximately half (45%) of participants not displaying any Z-band streaming post-RE, the Z-band streaming data violated assumptions of normality and were thus analyzed by linear mixed models analysis with group and measurement time as fixed factors and subjects as random factors. Values with two standard deviations from the mean were classified as outliers, and sensitivity analyses were performed with the outliers winsorized [47] to the next less extreme value. Effect size calculations were included to facilitate practical interpretations [48] but without the controversial practice of inferential statistics [49]. Therefore, Hedge’s g [50] was calculated to determine effect sizes and confidence intervals on the effect size for muscle strength and soreness, with the thresholds for small, moderate, and large effect sizes set at 0.2, 0.5, and 0.8, respectively. Area under the curve (AUC) was calculated over the 96 h post-RE period using the trapezoidal rule, and Pearson’s correlation coefficient was calculated to investigate relationships between post-exercise integrated MyoPS, MVC AUC, total perceived muscle soreness AUC, Z-band streaming AUC, muscle HSP25 AUC, muscle HSP72 AUC, plasma CK AUC, and 4 h plasma IL-6. Due to the impact of outliers on correlations [51], outliers were winsorized [47] to the next less extreme value prior to correlational analysis. Data were analyzed in SPSS statistics 24 (IBM, Armonk, NY, USA), with significance set at *p* < 0.05.

## 3. Results

### 3.1. Study Blinding

Due to potential placebo effects on performance and perceptual outcomes, it was important to blind participants to their supplement group. When asked to guess the test drink they received, participants had the option to choose from, “Protein”, “Carbohydrate”, or “Don’t know”, and then rated their confidence in their choice on a scale from 1 (not confident) to 5 (very confident). Out of the 20 participants, 8 correctly guessed the test drink they received, and 6 of these correct guesses were confident (3 or higher). We therefore believe that participants were well blinded, and that potential knowledge of their treatment condition was not a factor in study outcomes.

### 3.2. Integrated Myofibrillar Fractional Synthetic rate

**Figure 2:**
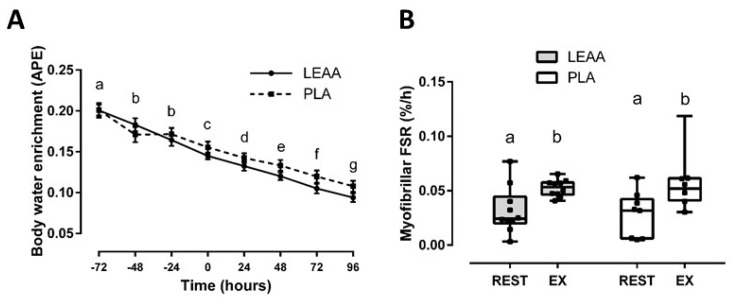
Mean (± SEM) body water enrichment over the 8 day exercise trial (**A**) and box-and-whisker plot of myofibrillar fractional synthetic rate before (REST) and during recovery from resistance exercise (EX) in response to LEAA or placebo consumption (**B**). Time points (i.e., hours, panel A; rest vs. exercise, panel B) with different letters are significantly different from each other (main effect for time, *p* < 0.05). APE: atom percent excess; FSR, fractional synthetic rate; EX, integrated 96 h post-exercise period; PLA, placebo.

Body water enrichment decayed over the 8 day trial (*p* < 0.001) and was not different between groups (*p* = 0.479; effect of interaction, *p* = 0.082; Figure 2). Integrated MyoPS over 96 h post-RE increased from baseline (*p* = 0.012) with no differences between groups (*p* = 0.982; effect of interaction, *p* = 0.531; Figure 2). Although a sensitivity analysis of outlier correction determined that post-RE MyoPS was numerically greater with LEAAs (mean ± SD; 0.053 ± 0.008) vs. placebo (0.050 ± 0.012), no significant between-group differences remained (*p* = 0.479).

### 3.3. Muscle Strength and Soreness

**Figure 3:**
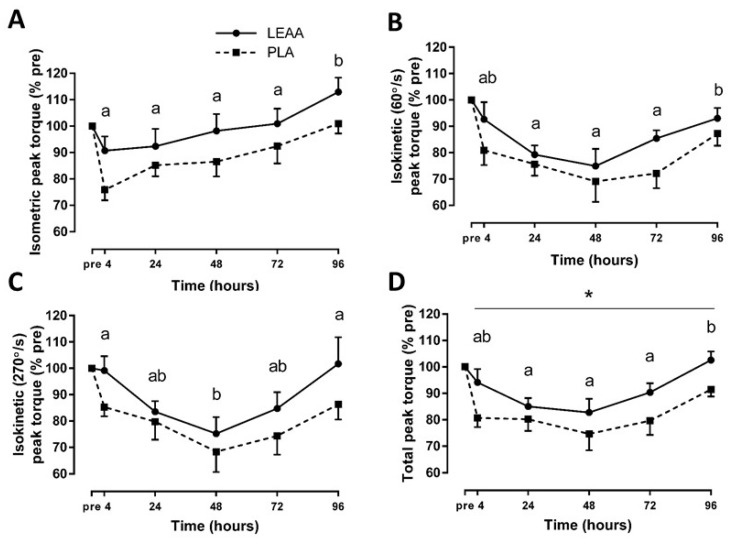
Mean (± SEM) isometric (**A**), isokinetic 60°/s (**B**), isokinetic 270°/s (**C**), and total (**D**) peak torque after resistance exercise in response to LEAA or placebo consumption. * Significant effect of group (*p* < 0.05). Time points with different letters are significantly different from each other (main effect for time, *p* < 0.05). Pre, pre-exercise; PLA, placebo.

There were no baseline group differences for mean (±SD) isometric (218.7 ± 40.7 vs. 243.8 ± 32.9 for LEAA and placebo groups, respectively; *p* > 0.05), slow isokinetic (163.1 ± 32.3 vs. 180.9 ± 34.4 for LEAA and placebo groups, respectively; *p* > 0.05), fast isokinetic (98.4 ± 16.8 vs. 105.1 ± 13.8 for LEAA and placebo groups, respectively; *p* > 0.05), and total (480.1 ± 76.8 vs. 529.8 ± 69.9 for LEAA and placebo groups, respectively; *p* > 0.05) peak torque (expressed in N∙m). Expressed as a percent of baseline, all MVC measures significantly changed throughout the 8 day trial in both groups (*p* < 0.05; Figure 3). There was a trend toward group differences in isometric (*p* = 0.092), slow isokinetic (*p* = 0.095) and fast isokinetic (*p* = 0.138) peak torque, and total peak torque significantly differed between groups (main effect of group, *p* = 0.042), with that of the LEAA group being greater than that of the placebo group. There were no interaction effects for all MVC measures (*p* > 0.05). Notable results from sensitivity analyses of outlier correction include a larger effect of group on isometric peak torque (*p* = 0.078) and total peak torque (*p* = 0.036) with that of the LEAA group being greater than that of the placebo group. There were overall moderate to large effects of LEAAs compared to placebo on post-RE MVC measures (Table 2).

**Figure 4:**
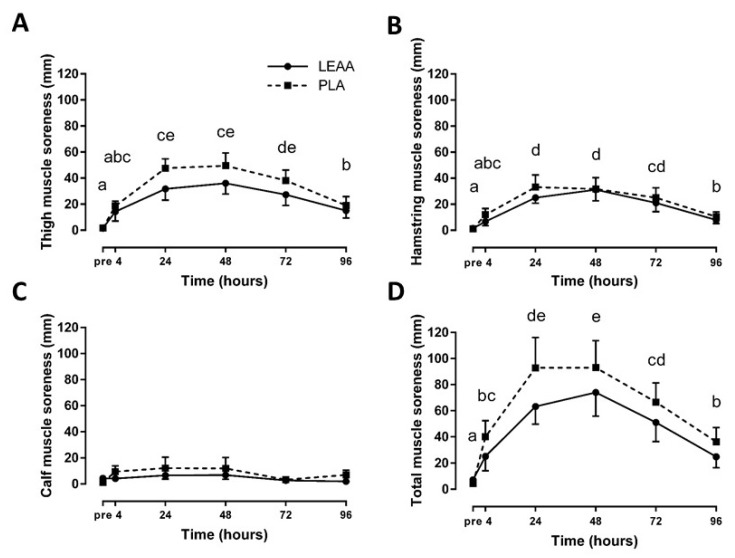
Mean (± SEM) thigh (**A**), hamstring (**B**), calf (**C**), and total (**D**) ratings of perceived muscle soreness after resistance exercise in response to LEAA or placebo consumption. Time points with different letters are significantly different from each other (main effect for time, *p* < 0.05). Pre, pre-exercise; PLA, placebo.

Thigh, hamstring, and total perceived muscle soreness significantly increased over the 8 day trial (*p* < 0.01), whereas calf soreness remained unchanged (*p* > 0.05; Figure 4). There were no significant group or interaction effects for all measures of perceived muscle soreness (*p* > 0.05), but there were small to moderate effect sizes favouring LEAAs over placebo (Table 3).

### 3.4. Z-band Streaming

**Figure 5:**
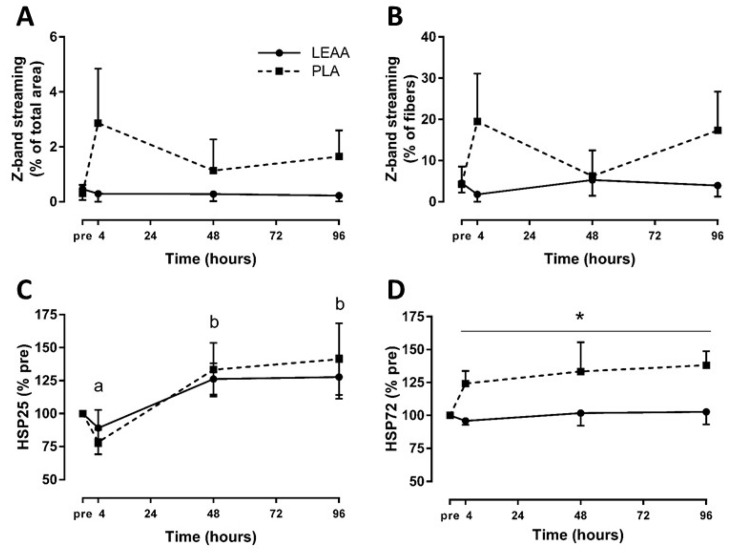
Mean (± SEM) Z-band streaming percentage area (**A**), percentage of fibers with Z-band streaming (**B**), and muscle heat shock protein 25 (**C**) and 72 (**D**) after resistance exercise in response to LEAA or placebo consumption. * Significant effect of group (*p* < 0.05). Time points with different letters are significantly different from each other (main effect for time, *p* < 0.05). Pre, pre-exercise; PLA, placebo.

The scores of percentage of muscle fiber area with Z-band streaming and percentage of muscle fibers with any Z-band streaming are presented in Figure 5. Although there was no effect of time on Z-band streaming percentage area and percentage of fibers with Z-band streaming (*p* = 0.557 and *p* = 0.639, respectively), there was a trend for placebo to be greater than LEAAs for both measures (main effect of group, *p* = 0.074 and *p* = 0.080, respectively).

### 3.5. Muscle Heat Shock Protein Response

Expressed as a percent of baseline, muscle HSP25, but not muscle HSP72 (*p* = 0.437), significantly increased over the 8 day trial (*p* < 0.001). Although muscle HSP25 was not significantly different between groups (*p* = 0.668), muscle HSP72 was significantly lower with LEAAs relative to placebo (main effect of group, *p* = 0.038; Figure 5). There were no interaction effects (*p* > 0.05). Notable results from sensitivity analyses of outlier correction include a larger effect of time (*p* = 0.008), group (*p* = 0.020), and interaction (*p* = 0.097) on muscle HSP72 for LEAAs to be lower than placebo.

### 3.6. Plasma Creatine Kinase and Interleukin-6

**Figure 6:**
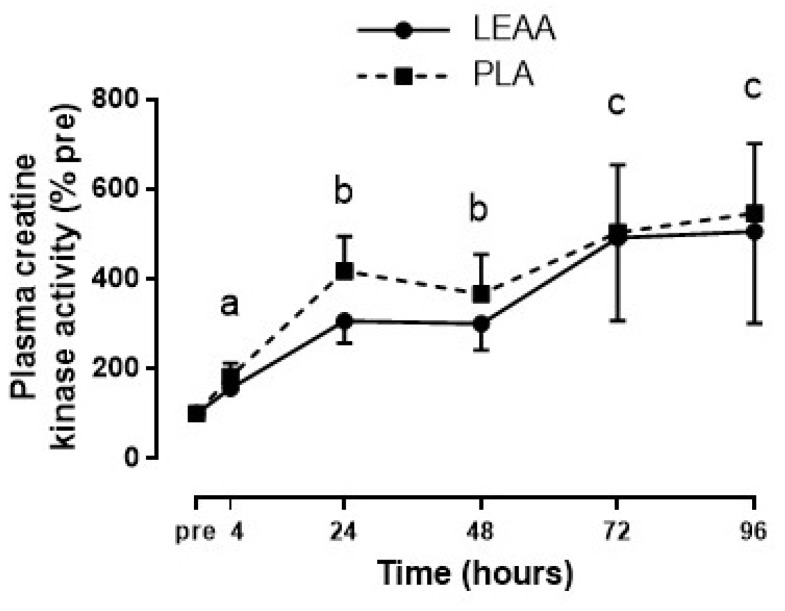
Mean (± SEM) plasma creatine kinase activity over 96 h after resistance exercise in response to LEAA or placebo consumption. Time points with different letters are significantly different from each other (main effect for time, *p* < 0.05). Pre, pre-exercise; PLA, placebo.

Expressed as a percent of baseline, plasma CK activity significantly increased over the 8 day trial (*p* = 0.029), but there were no effects of group or interaction (*p* > 0.05; Figure 6). Plasma IL-6 concentrations at pre-exercise, 24, 48, 72, and 96 h were all below the lower limit of reliable quantification (3.1 pg/mL) and were excluded in the analysis. Therefore, an independent samples t−test was conducted to analyze 4 h plasma IL-6 between groups, which revealed higher mean (± SD) post-RE plasma IL-6 concentrations with LEAAs (42.6 ± 43.5) compared to placebo (17.7 ± 17.7) that was not statistically significant (*p* = 0.130). Notable results from sensitivity analyses of outlier correction include a larger effect of time (*p* = 0.004), group (*p* = 0.206), and interaction (*p* = 0.261) on plasma CK for LEAAs to be lower than placebo, and a larger effect of group (*p* = 0.053) on plasma IL-6 for LEAAs to be higher than placebo.

### 3.7. Correlational Analysis

Figures for the correlative data presented here can be found in Supplementary Materials S1. MyoPS, Z-band streaming, and plasma CK did not significantly correlate with any variables (*p* > 0.05). Total muscle soreness correlated with isometric (*p* = 0.001, r = −0.701), fast isokinetic (*p* = 0.045, r = −0.452), and total (*p* = 0.006, r = −0.592) peak torque. Muscle HSP25 and HSP72 only correlated with each other (*p* = 0.037, r = 0.481). Plasma IL-6 only correlated with fast isokinetic peak torque (*p* = 0.046, r = 0.476). Aside from correlations within MVC measures, no other correlations were apparent for all variables. 

## 4. Discussion

The main finding of this study was that, in contrast to our hypothesis, LEAA supplementation three times per day was not more effective than placebo at enhancing integrated MyoPS over 96 h following an unaccustomed bout of RE in recreationally active men consuming a controlled diet with 1.2 g/kg/d of protein. However, consistent with our hypothesis, LEAAs moderately improved recovery from post-RE muscle damage, which interestingly did not correlate with changes in MyoPS. 

### 4.1. Effect of LEAAs on Integrated Myofibrillar Protein Synthesis

Resistance exercise increased integrated rates of MyoPS over the 96 h post-RE recovery period in our recreationally active young men. This is consistent with the ability of RE to stimulate MyoPS for at least 48 h [2,52] and perhaps as long as 72 h [53]. Although integrated rates of MyoPS increased in the 96 h post-RE recovery period in the present study, LEAA supplementation did not enhance the effect of exercise on MyoPS as compared to an isocaloric placebo. Traditional primed-constant infusion studies have demonstrated that the post-exercise ingestion of dietary amino acids is essential to maximize MyoPS during the early (i.e., <4 h) recovery period [46,54]. It has been suggested that the equivalent of ~1.0−2.0 g of leucine may be required to enhance post-exercise rates of MyoPS in young men of average body mass [46,54], which suggests that our dose of LEAAs (1.6 g leucine/drink) would have been sufficient to enhance MyoPS if provided as a single acute meal within a controlled laboratory based setting. Unfortunately, we were not able to determine the effect of LEAAs on 4 h post-RE MyoPS using D_2_O. We speculate that this may be related to our lower D_2_O dosing protocol, which was based on previous seminal work [3] but has subsequently been modified to a higher dose (i.e., ~400 mL vs. the current 150 mL 70atom% D_2_O) for measurements over shorter incorporation times [43,55]. Nevertheless, the lack of effect of thrice daily LEAAs on integrated MyoPS suggests that other factors within the free-living environment (e.g., habitual activity, dietary protein intake, etc.) may impact the ability to effectively translate acute amino acid supplement studies using primed-constant infusions outside of a traditional laboratory setting.

To increase the ecological validity of the study, participants in the present study consumed a controlled diet of 1.2 g/kg/d, which is slightly lower than the North American average [37] but is in line with recent suggested intakes for recreationally active males [38] and at the lower end of consensus recommendations for athletes [56]. However, we provided the diet in equally spaced meals in keeping with recommendations to optimize protein dose [57,58] and distribution [59], which may have elicited high integrated MyoPS rates in both groups. For example, although participants were instructed to separate test drinks and meals by at least 1.5 h, the combination of RE increasing dietary amino acid efficiency [41] and the ability of a moderate protein−containing meal to sustain MyoPS for greater than 3 h [59,60] could have diminished the anabolic potential of LEAAs. Alternatively, it is possible that a greater intrameal EAA intake (i.e., 15 g, which contained 2.79 g of leucine) is required to enhance MPS in otherwise adequately fed recreationally active individuals [61]. This could be consistent with the ability of 5 g of leucine supplemented to meals to be sufficient to enhance post-RE MyoPS rates in free-living older men when consuming a low (0.8 g/kg/d) or higher protein (1.2 g/kg/d) intake [19]. However, it should be acknowledged that the greater anabolic potential of leucine in these older adults could be related in part to the greater per meal protein requirement to maximize MyoPS in older adults [57,62], which could have increased the anabolic potential of leucine supplementation in these studies that otherwise provided suboptimal daily protein intake for older adults. Therefore, it is possible that post-RE MyoPS rates were not greater with LEAAs in the present study because participants were already receiving an optimal dose and distribution of daily protein from their controlled diet.

### 4.2. Effect of LEAAs on Muscle Damage

Studies investigating protein supplementation and muscle damage recovery following lower-body RE in untrained individuals have yielded inconsistent findings. For example, studies have reported positive effects of protein supplementation on post-RE muscle soreness but not muscle strength [30], muscle strength but not muscle soreness [31,63], or neither [64]. The variability may be explained by differences in protein supplement type (whey protein, milk protein, or BCAAs), supplementation timing (before exercise, after exercise, multiple times per day), exercise protocol, overall macronutrient intake, and/or the overreliance on parametric statistics and lack of effect size analysis [48]. In the present study, effect size analysis revealed that LEAAs had small to moderate effects on reducing muscle soreness, and moderate to large effects on preserving muscle torque production that reached statistical significance when peak torque measures were summated. These findings are consistent with a recent meta-analysis [65] reporting that protein supplementation seems to have overall moderate effects on reducing muscle function losses in the days following RE.

The underlying mechanism for the attenuation of muscle force losses with LEAA supplementation is unknown. Previous authors have suggested that the greater bioavailability of amino acids, particularly the BCAAs, may reduce the extent of myofibrillar breakdown following eccentric exercise and thus preserve muscle function [66,67]. However, given the relatively slow rate of myofibrillar protein turnover [68], any potential anti-catabolic effect of LEAAs on myofibrillar protein, if it were indeed to influence maximal strength, would likely only influence muscle function later (i.e., >4 h) in recovery. Nevertheless, it is also unclear whether the greater muscle torque with LEAAs may be indirectly related to attenuations in exercise-induced muscle soreness given the relationship we observed between these variables. We cannot discount the possibility that LEAAs supported greater myofibrillar remodeling at particular stress points within myofibres and/or their associative contractile elements. Finally, lateral transmission of force between the sarcomere to the collagen extracellular matrix (ECM) is important for and precedes its transmission to the bone for muscle torque production [69,70]. Emerging evidence suggests that tendon collagen remodeling with exercise may be enhanced with leucine-enriched nutritional supplements [71,72]. Given that LEAAs may induce a small (~30%) non-significant increase in muscle collagen synthesis during recovery from damaging exercise in rodents [11], we cannot discount the possibility that LEAAs had a small benefit for muscle torque production in the present study via enhanced remodeling of the muscle ECM. Ultimately, the mechanism by which LEAA/leucine/protein supplementation mitigates muscle force losses warrants additional investigation.

Damaging eccentric exercise elevates the content of HSP25 (also known as HSP27 in humans) and HSP72 in skeletal muscle [24,73,74]. Muscle HSP25 is thought to play a role in stabilizing denatured proteins, whereas muscle HSP72 prevents unfavourable aggregation of partially folded proteins and facilitates correct folding of polypeptides during synthesis [23]. Presumably, muscle HSP72 would be upregulated to support increases in post-RE MyoPS. However, in the present study, although MyoPS increased similarly in both LEAA and placebo groups following RE, muscle HSP72 remained at baseline levels with LEAAs but significantly rose with placebo. Considering that LEAAs also partially reduced the appearance of damage to the myofibril ultrastructure as evaluated by Z-band streaming, these data suggest that the greater induction of muscle HSP72 in the placebo group was directed toward repairing a larger magnitude of intracellular protein damage. Conversely, a recent study in healthy older adults reported increases in post-exercise HSP72 with milk protein supplementation [75]. A potential explanation for the discrepancy is that, unlike milk protein, LEAAs do not contain the non-essential amino acid glutamine that can independently enhance HSP expression [76]. Additional research is required to determine the nutritional regulation of the HSP response after eccentric exercise and its relevance to objectively measured alterations in sarcomeric structure (e.g., Z-band streaming).

The present study employed a parallel-group design to assess the impact of LEAAs on post-RE muscle damage in order to avoid the prohibitively long washout that would otherwise be required to allow for an attenuation of the repeated bout effect, which might persist for up to 12 weeks [77]. In addition, our interest in concurrently measuring both muscle performance and MyoPS without either objectives influencing the other dictated that we perform a bilateral exercise stimulus, which rendered the unilateral exercise crossover model untenable. Although within-participant study designs may be statistically more robust and limit interindividual physiological and/or perceptual variability, parallel group designs are far more prevalent in studies investigating the impact of amino acid-based interventions on exercise-induced muscle damage given the challenges with the repeated bout effect [12,29,30,31,32,63,64]. Therefore, although it is possible that we were able to effectively employ a within-participant cross-over design, the small to moderate effect sizes in favour of LEAAs for some secondary outcomes (e.g., muscle strength and soreness) could have reached statistical significance. In other words, despite the use of a statistically, and potentially biologically, inferior study design, we nevertheless did observe significance in our main secondary outcome of total muscle strength. We interpret this as being physiologically relevant for individuals aiming to minimize the effects of exercise-induced muscle damage, particularly when viewed in parallel with the generally consistent small to moderate effects sizes in favour of LEAAs for the other secondary outcomes. 

### 4.3. Correlation between MyoPS and Muscle Damage Markers

It has been suggested that increased MyoPS following unaccustomed RE and protein ingestion facilitates enhanced skeletal muscle remodeling and therefore may improve the recovery from exercise-induced muscle damage [27], but this has little empirical evidence. In the present study, we found no relationship between MyoPS and any markers of muscle damage, which is at odds with the purported role for myofibrillar remodeling to repair damaged myofibrils [27]. However, our findings would be congruent with those of Damas et al. [2], who reported no relationship between MyoPS and Z-band streaming, MVC, perceived muscle soreness, or plasma CK following a single bout of RE in untrained males. Nevertheless, muscle protein turns over at ~1%−2%/day, is highly variable between individuals [78], and may be greatest at localized areas of myofibrillar remodeling [22], which may have precluded our ability to determine any relationship between MyoPS (of all sampled fibers) and muscle damage or function. Finally, it is important to consider that muscle strength has a substantial neuromuscular component that, while potentially influenced by muscle-specific factors (e.g., Z-band streaming, muscle inflammation), is significantly impacted by extramuscular factors (e.g., motor unit activation, muscle fiber recruitment, etc.) [79]. Therefore, under the conditions of the present study, it does not seem that greater rates of MyoPS are associated with superior muscle damage recovery following RE in recreationally active males.

## 5. Conclusions

In summary, the increase in integrated MyoPS over 96 h post-RE was not enhanced by thrice daily LEAA supplementation (containing 1.6 g leucine each) compared to placebo. However, LEAAs preserved muscle force production and moderately attenuated muscle soreness, which was accompanied by a reduced muscle HSP72 response and a trend toward decreased Z-band streaming. Although muscle soreness and strength were correlated, no relationship was observed between changes in MyoPS and any markers of muscle damage. Collectively, our data suggest that LEAAs have the ability to mitigate muscle damage in the days following an acute bout of resistance exercise independent of augmenting free-living MyoPS. Future research examining the mechanisms of inflammation and muscle damage recovery in response to amino acid supplementation and unaccustomed exercise is warranted.

## Figures and Tables

**Table 1:** Participant characteristics.

	LEAAs (*n* = 10)	Placebo (*n* = 10)
Age (years)	24 ± 3.7	23 ± 4.9
Fat-free mass (kg)	63.6 ± 10.1	63.6 ± 5.3
RMR (kcal/day)	1655 ± 275	1684 ± 143
Leg press 1RM (kg)	255.5 ± 39.5	257.5 ± 48.3
Leg extension 1RM (kg)	106.4 ± 28.2	106.4 ± 26.4
Habitual dietary protein (g/kg/day)	1.598 ± 0.605	1.412 ± 0.704
Habitual dietary energy (kcal/day)	2457 ± 1230	2391 ± 1148
Trial dietary protein ^1^ (g/kg/day)	1.198 ± 0.005	1.173 ± 0.043
Trial dietary energy ^1^ (kcal/day)	2646 ± 448.5	2619 ± 264.3
Trial TEE ^1^ (kcal/day)	678 ± 258	579 ± 197
Trial MVPA ^1^ (min/day)	93.0 ± 29.4	80.9 ± 27.2
Trial steps ^1^ (count/day)	9071 ± 2466	7511 ± 2839

Values are the mean ± SD. RMR, resting metabolic rate; 1RM, one repetition maximum; TEE, total energy expenditure; MVPA, moderate to vigorous physical activity; LEAAs, leucine-enriched essential amino acids. ^1^ Measured during the 8 day exercise trial.

**Table 2:** Effect sizes and confidence intervals for isometric, slow isokinetic, fast isokinetic, and total peak torque after resistance exercise in response to LEAA versus placebo consumption. Thresholds for small, moderate, and large effect sizes are 0.2, 0.5, and 0.8, respectively.

Measurement	Time	Hedge’s g (LEAAs vs. Placebo)	95% CI on Hedge’s g
Isometric peak torque (% pre)	4 h	0.95	0.05–1.91
	24 h	0.40	−0.48–1.29
	48 h	0.59	−0.29–1.51
	72 h	0.42	−0.46–1.32
	96 h	0.77	−0.12–1.71
Isokinetic (60°/s) peak torque (% pre)	4 h	0.58	−0.30–1.50
	24 h	0.28	−0.59–1.17
	48 h	0.25	−0.63–1.13
	72 h	0.89	−0.01–1.85
	96 h	0.41	−0.46–1.31
Isokinetic (270°/s) peak torque (% pre)	4 h	0.91	0.01–1.86
	24 h	0.21	−0.67–1.09
	48 h	0.30	−0.57–1.19
	72 h	0.47	−0.40–1.38
	96 h	0.57	−0.31–1.48
Total peak torque (% pre)	4 h	0.95	0.03–1.90
	24 h	0.40	−0.50–1.28
	48 h	0.59	−0.45–0.33
	72 h	0.42	−0.17–1.65
	96 h	0.77	0.19–2.10

**Table 3:** Effect sizes and confidence intervals for thigh, hamstring, calf, and total perceived muscle soreness after resistance exercise in response to LEAA versus placebo consumption. Thresholds for small, moderate, and large effect sizes are 0.2, 0.5, and 0.8 respectively.

Measurement	Time	Hedge’s g (LEAAs vs. Placebo)	95% CI on Hedge’s g
Thigh muscle soreness	4 h	−0.23	−1.12–0.65
	24 h	−0.60	−1.52–0.28
	48 h	−0.45	−1.35–0.43
	72 h	−0.41	−1.31–0.47
	96 h	−0.19	−1.07–0.69
Hamstring muscle soreness	4 h	−0.40	−1.30–0.48
	24 h	−0.34	−1.24–0.53
	48 h	−0.02	−0.89–0.86
	72 h	−0.17	−1.05–0.71
	96 h	−0.22	−1.11–0.65
Calf muscle soreness	4 h	−0.47	−1.38–0.41
	24 h	−0.27	−1.16–0.60
	48 h	−0.24	−1.13–0.63
	72 h	−0.10	−0.98–0.78
	96 h	−0.56	−1.47–0.32
Total muscle soreness	4 h	−0.38	−1.29–0.49
	24 h	−0.47	−1.38–0.40
	48 h	−0.29	−1.19–0.58
	72 h	−0.32	−1.21–0.56
	96 h	−0.36	−1.25–0.52

## Supplementary Materials

The following are available online at https://www.mdpi.com/2072-6643/12/4/1061/s1, Figure S1: Linear correlations for select post-resistance exercise outcomes.
