# Supplementary material for: Leucine-Enriched Essential Amino Acids Improve Recovery from Post-Exercise Muscle Damage Independent of Increases in Integrated Myofibrillar Protein Synthesis in Young Men
Source: Nutrients. 2020 Apr 11;12(4):1061. doi: 10.3390/nu12041061 (PMC7231404; doi:10.3390/nu12041061)
Supplement: Supplementary file 1 [file nutrients-12-01061-s001.pdf]

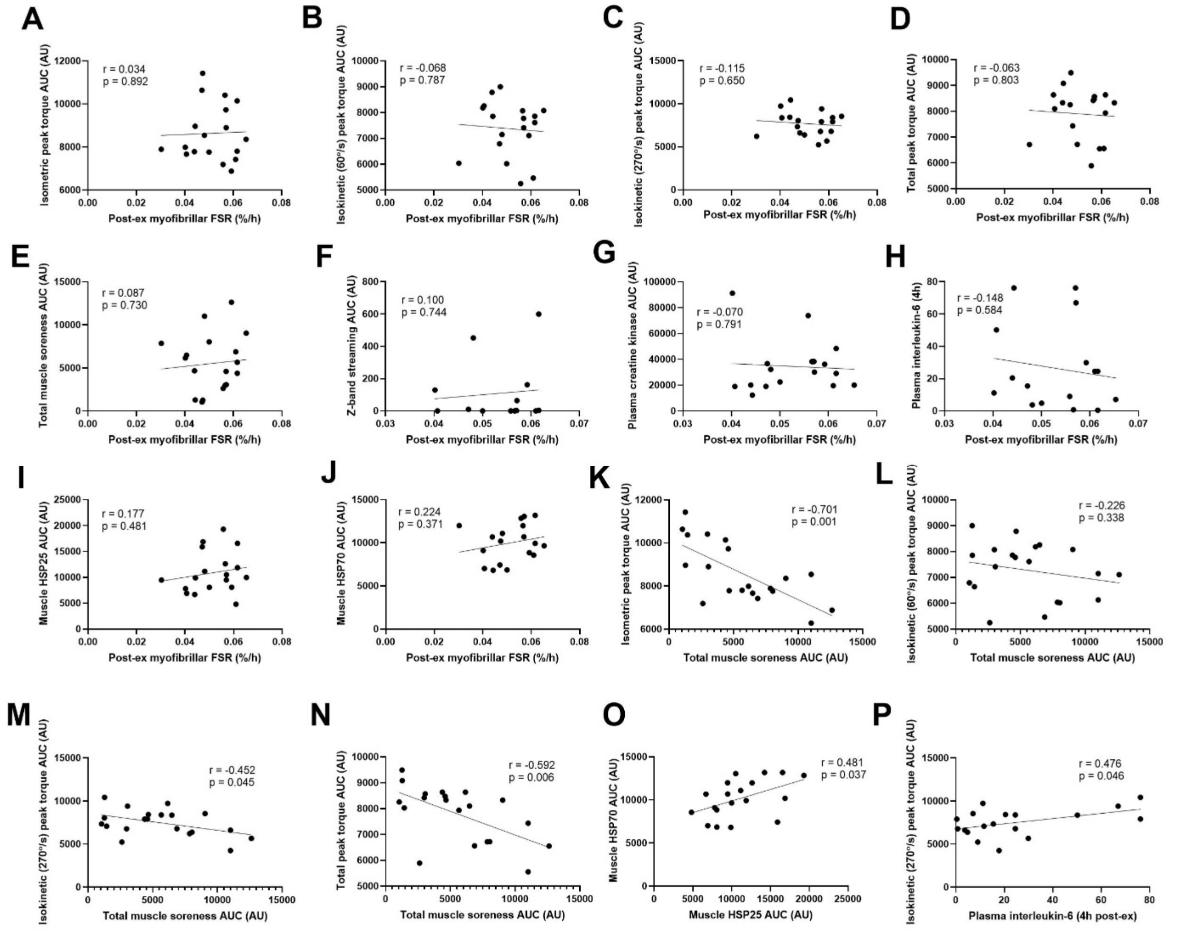

Supplementary Material S1. Linear correlations for select post-resistance exercise outcomes, including linear correlations between myofibrillar fractional synthetic rate and isometric peak torque (A), isokinetic 60°/s peak torque (B), isokinetic 270°/s peak torque (C), total peak torque (D), total muscle soreness (E), Z-band streaming (F), plasma creatine kinase activity (G), plasma interleukin-6 (H), muscle heat shock protein 25 (I), and muscle heat shock protein 70 (J), linear correlations between total muscle soreness and isometric peak torque (K), isokinetic 60°/s peak torque (L), and isokinetic 270°/s peak torque (M), and total peak torque (N), and linear correlations between muscle heat shock protein 25 and muscle heat shock protein 70 (O) and between plasma interleukin-6 and isokinetic 270°/s peak torque (P). Post-ex, 96h post-resistance exercise period; AUC, area under the curve; AU, arbitrary units.
